# Endoglin (CD105) and VEGF as potential angiogenic and dissemination markers for colorectal cancer

**DOI:** 10.1186/s12957-020-01871-2

**Published:** 2020-05-20

**Authors:** Ana Nogués, Eunate Gallardo-Vara, Mª. Paz Zafra, Paloma Mate, Jose Luis Marijuan, Alfredo Alonso, Luisa Mª. Botella, Mª. Isabel Prieto

**Affiliations:** 1 Department of General Surgery, Hospital Universitario La Paz, 28046 Madrid, Spain; 2 Centro de Investigaciones Biológicas (CIB), Consejo Superior de Investigaciones Científicas (CSIC), Centro de Investigación Biomédica en Red de Enfermedades Raras (CIBERER), 28040 Madrid, Spain; 3 Department of Medicine, Sandra and Edward Meyer Cancer Center, Weill Cornell Medicine, New York, NY 10021 USA; 4 Department of General Surgery, Hospital Universitario del Sureste de Madrid, Arganda del Rey, Madrid, Spain

**Keywords:** Endoglin (CD105), Soluble Endoglin, VEGF, Colorectal cancer (CRC), Tumor angiogenesis, Biomarkers

## Abstract

**Background:**

Colorectal cancer (CRC) is an important current problem concerning public health due to its high incidence and mortality. Advances in molecular and cellular knowledge and the detection of new disease biomarkers are very important to improve prognosis, prediction, and early diagnosis. In this study, we aimed to analyze the gene and protein expression levels of two angiogenic markers, VEGF and soluble Endoglin, during different tumor stages as well as at different stages of cancer treatment, to predict the diagnosis and evolution of colon and rectal cancer.

**Material and methods:**

This study includes 133 CRC patients (93 with colon cancer and 40 with rectal cancer) on which the gene and protein expression of Endoglin (membrane and soluble form) and VEGF were analyzed by molecular and immunohistochemical techniques on different tumor stage samples and plasma obtained preoperatively as well as 3, 6, and 9 months after resection of the tumor.

**Results:**

VEGF and Endoglin gene expressions were higher in tumor tissue than in surrounding non-tumoral tissue for both types of cancer. The VEGF levels in plasma were found to decrease in less aggressive tumors, whereas soluble Endoglin was increased in preoperative samples of patients with metastasis. Membrane Endoglin expression was higher on the vascular endothelium of more aggressive tumors. In contrast, Endoglin expression was mainly in the colon epithelium in less aggressive stage tumors.

**Conclusion:**

Endoglin and VEGF are proteins with a major role in the tumor angiogenesis process. This study performed with a wide cohort of human samples shows that both proteins seem to be valuable biomarkers in the diagnosis and prognosis of CRC.

## Introduction

Colorectal cancer (CRC) is the second leading cause of cancer-related death in developed countries and is thus a significant clinical problem [1].

CRC is the result of an accumulation of genetic alterations, either inherited and/or caused by endogenous and exogenous factors that transform the normal tissue to benign adenoma/dysplasia to malignancy [2–6].

Understanding the molecular changes occurring in CRC is important to discover potential new biomarkers that help to improve the early detection, prognosis, and prediction of response to treatment [7, 8]. The identification of such biomarkers might lead to more personalized, specific, and less toxic treatments for CRC patients. The angiogenesis process, involving the formation of new blood vessels from preexisting vessels, is essential for solid tumor growth, progression, and, most importantly, metastasis formation [9, 10]. Angiogenesis is highly regulated by various factors involved in different signalling pathways. Among these pathways, the Vascular Endothelial Growth Factor (VEGF) and Transforming Growth Factor-β (TGF-β) family of proteins are especially relevant [11, 12].

VEGF is one of the most potent angiogenic growth factors and is expressed by most human cancers. An increase in VEGF synthesis has been associated with tumor vascularization, metastasis, chemoresistance, and a worse prognosis [11, 13]. VEGF and its receptors are usually overexpressed in solid tumors and are promising targets for the treatment of neoplasms [14, 15].

Endoglin (Eng), also called CD105, is a 180-kDa homodimeric transmembrane glycoprotein that belongs to TGF-β family receptors. Endoglin is essentially expressed on endothelial cells that form the arteries, veins, and capillaries and plays an important role in cardiovascular diseases, angiogenic processes, and cancer [16–19]. Endoglin binds TGF-β1, TGF-β3, and BMP9 with great affinity, forming a receptor complex with TβR-I and TβR-II [20–22]. A soluble form of Endoglin (sEng) has been described. sEng is released through the proteolytic cleavage of the extracellular domain of membrane Endoglin by the metalloprotease MMP14 [23]. An increase in sEng in circulation occurs during different pathophysiological processes, such as endothelial injury, migration, angiogenesis, inflammation, cardiovascular diseases, preeclampsia, and tumor angiogenesis [24–27].

The main objective of this work is to determine VEGF and Endoglin expression in tumoral and non-tumoral adjacent tissue of the surgical pieces and in plasma, before and after surgery, to correlate VEGF and Endoglin expression in tumor samples with the clinical and pathological stages, progression, and prognosis in colorectal cancer.

## Materials and methods

### Patients

This longitudinal study prospectively included 133 patients with malignant polyps and different colorectal cancer (CRC) stages (0–IV). We included pedunculated or sessile polyps with invasive carcinoma that fulfilled the criteria for surgery for any of these reasons: incomplete endoscopic resection, poorly differentiated lesions, vascular and/or lymphatic invasion, and those whose margin of resection was invaded. After the resection of tumor samples, the patients were followed-up for a maximum period of 2 years. All tissue samples were acquired, stored in a biobank (IdiPaz Biobank), and subsequently used in accordance with the Declaration of Helsinki and the requirements of current Spanish legislation on working with human biological samples and the protection of personal data.

All patients in the study fulfilled the inclusion criteria: older than 18 years, informed signed consent, specific blood count, colonoscopy with biopsy and thoracic-abdominal-pelvic computed tomography (CT) for the clinical diagnosis of colon cancer (CC), pelvic magnetic resonance (MR), and/or endoanal ultrasound scan for the clinical diagnosis of rectal cancer (RC). Colon and rectal cancer samples were classified in different stages according to the last 2017 edition of the TNM system of the American Joint Committee on Cancer (AJCC) and the Union for International Cancer Control (UICC) [8]. This study included variables, such as age, sex, tumor localization, TNM staging, surgical intervention, neoadjuvant or adjuvant therapy, existence, and localization of metastasis and exitus.

### ELISA of soluble Endoglin and VEGF

Blood samples were collected from 37 RC and 81 CC patients at time 0 (immediately before surgery) and at different time points (3, 6, and 9 months) after resection of the tumor sample. sEng and human VEGF concentrations in plasma were measured and quantified by ELISA, according to the manufacturer’s protocol by Quantikine Human Endoglin/CD105 and Quantikine Human VEGF, respectively (DNDG00 and DVE00; R&D Systems). All immunoassays were measured with a GloMax multidetection system (Promega).

### Immunohistochemistry

Endoglin expression was evaluated in paraffin-embedded tissue samples from extracted tumors at different CC and RC stages, and the control of healthy tissue in both cases, colonic or rectal cancer, was represented by a piece of normal tissue adjacent to the tumor lesion. The number of samples analyzed for CC was 43, 26 patients with less aggressive stages (malignant polyp and stages 0, I and II) and 17 patients with aggressive stages (stages III and IV). In the case of RC, there were only 5 samples (3 less aggressive and 2 more aggressive). Tumoral and non-tumoral pieces contained roughly the same proportion of isolated vessels; therefore, the normalization is quite accurate. We were very cautious not to take the highly vascularized regions of the tumor, to avoid the bias in the interpretation of the results. For the immunohistochemical staining of Endoglin, 5 μm deparaffinized and hydrated sections were incubated with a primary mouse monoclonal anti-CD105 antibody (clone SN6H, M3527, Dako). After primary antibody incubation, the samples were washed and incubated with HRP-conjugated secondary goat anti-mouse antibodies. HRP activity was amplified with DakoEnVision^TM^ + Dual Link System-HRP (K4063, Dako). Visualization was performed with a DAB substrate Kit (K3467, Dako). Slides were counterstained with hematoxylin 0.02% and mounted with DPX (44581, Sigma-Aldrich). Images were taken with an Olympus digital camera coupled to an Axio Vert. A1 Zeiss microscope.

### Total RNA extraction, cDNA synthesis, and quantitative real-time PCR (RT-qPCR)

Total RNA was isolated from OCT-embedded biopsy samples with a SpeedTools Total RNA Extraction Kit (Biotools). The concentration of total RNA was assessed spectrophotometrically. In addition to the quantification of RNA, the purity was also measured by electropherogram to be sure about the quality of the samples and that they were DNA-free. One microgram of total RNA was reverse transcribed using a cDNA reverse transcription kit (4368814, Applied Biosystems); the priming strategy was rando primers. The resulting cDNA was used as a template for qPCR. The total amount of cDNA was 2 μL per qPCR reaction, corresponding to a 1/20 dilution of the original cDNA product. The following forward (Fw) and reverse (Rv) oligonucleotides were used for selected genes (VEGF, Eng): hEng, Fw 5′-GCCCCGAGAGGTGCTTCT-3′ and Rv 5′-TGCAGGAAGACACTGCTGT-3′; hVEGF, Fw 5′-TCTACCTCCACCATGCCAAGT-3′and Rv 5′-GCTGCGCTGATAGACATCCA-3′. The size of real time products was between 70 and 100 nt, depending on the gene. As an internal control, the mRNA levels of h18S were measured using the primers Fw 5′-CGCTCCACCAACTAAGAACG-3′ and Rv 5′-CTAACACGGGAAACCTCAC-3′. The samples were amplified using the iQSyBR-Green Supermix (Bio-Rad), and the amplicons were detected using a real-time PCR iQ5 instrument. The expression was calculated according to delta-delta Ct method, which is a standard broadly accepted method in literature [28].

### Statistical analysis

Paired analysis was made by *T* Student test; ANOVA was used for comparison of more than 2 groups. Degree of statistical significance was considered as follows: *p* value ≤ 0.05 (*), *p* value ≤ 0.01 (**), and *p* value ≤ 0.001 (***).

## Results

The 133 patients with CRC were divided into two groups, colon and rectal cancer, for the analysis. The patient characteristics are summarized in Tables 1 and 2.

**Table 1:** Colon cancer patient characteristics

Characteristic	Patients (*n* = 93)	Frequency (%)
Gender		
Male	58	62.4
Female	35	37.6
Age (years)		
≤ 65	24	25.8
> 65	69	74.2
Tumor site		
Right colon	31	33.3
Transverse colon	8	8.6
Descending colon	13	14
Sigmoid colon	40	43
Synchronous tumors	1	1.1
Colonoscopy biopsy		
Adenocarcinoma	76	81.7
Polyp	17	18.3
TNM postoperative stage (pTNM)		
Less aggressive (polyp, stages 0, I, and II)	56	60.2
More aggressive (stages III and IV)	37	39.8
Adjuvant treatment (XELOX, oxaliplatin-capecitabine)		
Yes	42	45.2
No	51	54.8
Metastasis		
Yes	21	22.6
Synchronous	12	12.9
Metachronous	9	9.7
No	72	77.4
Metastasis location		
Liver	14	15.1
Lung	2	2.1
Ganglion	1	1.1
Other locations	4	4.3
Survival (at the end of study)		
Alive	88	94.6
Died	5	5.4

*pTNM* pathological classification of tumor-node-metastasis system according to 2017 last edition

**Table 2:** Rectal cancer patient characteristics

Characteristic	Patients (*n* = 40)	Frequency (%)
Gender		
Male	26	65
Female	14	35
Age (years)		
≤ 65	15	37.5
> 65	25	62.5
Tumor site		
High rectum (11–15 cm from AM)	13	32.5
Medium or low rectum (6–10 cm and 0–5 cm from AM)	27	67.5
Colonoscopy biopsy		
Adenocarcinoma	39	97.5
Polyp	1	2.5
Neoadjuvant treatment (radiotherapy plus capecitabine)		
Yes	27	67.5
No	13	32.5
TNM preoperative stage (cTNM)		
Less aggressive (polyp, stages 0, I, and II)	14	35
More aggressive (stages III and IV)	26	65
TNM postoperative stage (pTNM/ypTNM)		
Less aggressive (polyp, stages 0, I, and II)	26	65
More aggressive (stages III and IV)	14	35
Adjuvant treatment (XELOX, oxaliplatin-capecitabine)		
Yes	28	70
No	12	30
Metastasis		
Yes	8	20
Synchronous	5	12.5
Metachronous	3	7.5
No	32	80
Metastasis location		
Liver	2	7.5
Lung	3	5
Ganglion	1	2.5
Other locations	2	5
Survival (at the endo of study)		
Alive	36	90
Died	4	4

*cTNM* clinical staging, *pTNM* pathologic staging, *ypTNM* neoadyuvant pathologic staging classifications of tumor-node-metastasis system according to 2017 last edition, *AM* anal margin

### Clinical description of the colon and rectal cancer cohorts

A total of 93 patients with CC were included in this study (Table 1). The preoperative stage was divided into two groups: 56 patients (60.2%) were diagnosed with less aggressive stages (malignant polyp and stages 0, I, and II), and 37 (39.8%) patients had more aggressive stages (stages III and IV). Adjuvant treatment was administered to 42 patients (45.2%) with stage III and stage II high risk as lymphovascular invasion or pathologic T4 tumors (pT4), according to the XELOX (oxaliplatin-capecitabine) scheme.

The group with RC comprised 40 patients (Table 2). The preoperative stage was divided into two categories: less aggressive (polyp and stages 0, I, and II), including 14 patients (35%), and more aggressive (stages III–IV), including 26 patients (65%). Before the surgery, 27 patients (67.55%) received neoadjuvant therapy consisting of radiotherapy plus capecitabine. The adjuvant treatment was administered to 28 patients (70%) according to the XELOX (oxaliplatin-capecitabine) scheme.

### Gene expression of Endoglin and VEGF in colon and rectal tissue samples

**Fig. 1:**
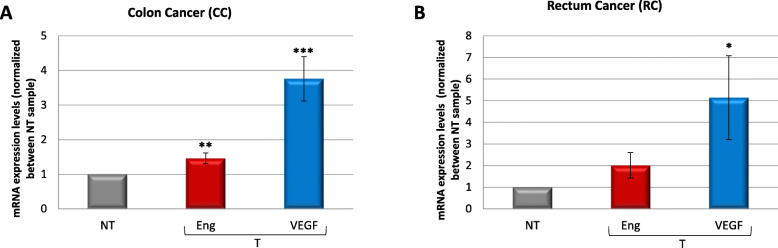
Endoglin and VEGF mRNA expression levels increase in tumor samples comparing with non-tumoral ones. Samples from tumoral and non-tumoral regions from different patients were collected during surgery. RNA was extracted and processed for RT-qPCR of human Endoglin and VEGF genes in colon cancer samples (**a**) and rectum cancer samples (**b**). Fold change of mRNA expression levels with respect to levels from non-tumoral samples are indicated. Results were normalized to 18SrRNA as a housekeeping gene, and respect to non-tumoral sample, reason why this value is the same for both genes. *p < 0.05; **p < 0.01; ***p < 0.001 with respect to non-tumoral gene expression. NT, non-tumoral; T, tumoral; Eng, Endoglin; VEGF, Vascular Endothelial Growth Factor

An increase in Endoglin and VEGF gene expression was found in CC samples compared with normal tissue samples (Fig. 1a), although no significant differences in Endoglin or VEGF gene levels were achieved across all tumor stages. Unfortunately, the variability is highly influenced by the type of treatment in more aggressive stages (stages III and IV), added to a low number of samples obtained, which makes it difficult to have statistically significant differences. Therefore, all tumor stages were included in a single group. The increment of VEGF expression is stronger than that of Endoglin: approximately 80% of tumors express VEGF with higher values than control, at variance with 60% of the tumors expressing higher Endoglin levels than control. On the other hand, although the number of samples with RC was smaller, Endoglin and VEGF gene expression levels were also quantified (Fig. 1b). VEGF gene expression was significantly higher (*p* < 0.05) in tumor tissue than in normal tissue. Endoglin gene expression was increased in tumors, but the differences were not statistically significant.

### Soluble Endoglin (sEng) and VEGF plasma levels in patients with colon cancer

**Fig. 2:**
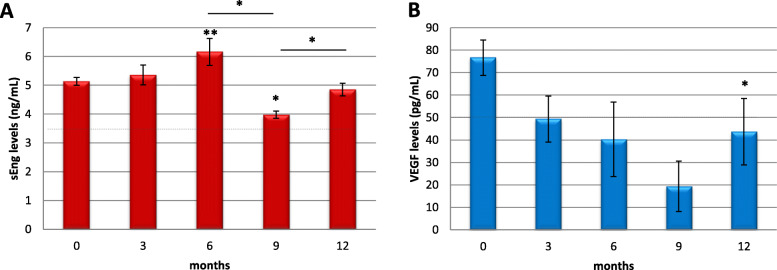
Soluble Endoglin and VEGF plasma levels change after resection of colon tumor sample. Plasma samples from different patients at time 0 and at different time points (3, 6, 9, and 12 months) after resection of tumor samples were collected, and levels of sEng in nanograms per milliliter (**a**) and VEGF in picograms per milliliter (**b**) were measured by ELISA and compared to time 0. The number of samples was different between each time point: *n* = 53 (0), *n* = 19 (3), *n* = 13 (6), *n* = 4 (9), *n* = 8 (12) for sEng determination and *n* = 74 (0), *n* = 22 (3), *n* = 15 (6), *n* = 4 (9), *n* = 13 (12) for VEGF determination. The dashed lines in both graphs indicate the considered normal values levels for each protein. *p < 0.05; **p < 0.01 with respect to time 0 unless indicated between specified conditions. sEng, soluble Endoglin; VEGF: Vascular Endothelial Growth Factor

sEng and VEGF levels were measured in patient plasma samples before surgery (time 0) and in a prospective analysis at 3, 6, and 9 months after tumor resection (Fig. 2). Considering soluble Endoglin values (Fig. 2a), in the cohort studied, the average was higher than the control values according to the literature (≤ 3.5 ng/mL) [29]. The experiments leading to the control values of the population of around 3.5 ng/mL for sEng were done in the same laboratory and by the some experts who have now performed the tests reported in the present research article. Although there were no statistically significant differences in sEng levels at any time point during the follow-up period, an increasing trend at up to 6 months after surgery was observed. In contrast, a decrease was observed in the last two time points and was significant after 9 months of tumor sample resection.

In less aggressive CC stages, sEng levels increase significantly up to 3–6 months after surgery, and then decrease thereafter. On the other hand, in more aggressive stages, sEng levels do not change during the follow-up period and are increased in all stages (Sup Figure 1A). The variability found in postoperative sEng levels could be due to the influence of treatment or non-treatment after surgery. Regarding treatment with bevacizumab, we only registered nine patients, but we have hypothesized that if the patient is a good responder, then sENG is difficult to detect, because angiogenesis decreases, and so is sENG and VEGF. However, if the patient becomes resistant to bevacizumab in time, the tumor will recur, and there will be neoangiogenesis again, with an increase in sEng and VEGF.

In relation to VEGF determinations, a threshold above 50 pg/mL could be assumed as pathological, while values in the range between 30 and 50 pg/mL may be considered the normal range. These data come from the ELISA test protocol references and can be considered normal according to the reviewed literature [30, 31]. The mean VEGF levels at time 0 were higher than the control values (30–50 pg/mL), although this value decreases in time and is maintained at the borderline of levels during the follow-up period (Fig. 2b).

Considering the TNM stage (Sup Figure 1B), at time 0, 58%, and 67% of patients with less or more aggressive phenotypes of CC, respectively, have more than 30–50 pg/mL serum VEGF, although there is no statistically significant difference. Notably, after resection of the tumor sample, patients with less aggressive tumors showed a gradual decrease in VEGF levels over months, while more aggressive tumors maintained higher than normal values after 6 months, and then decreased thereafter.

**Fig. 3:**
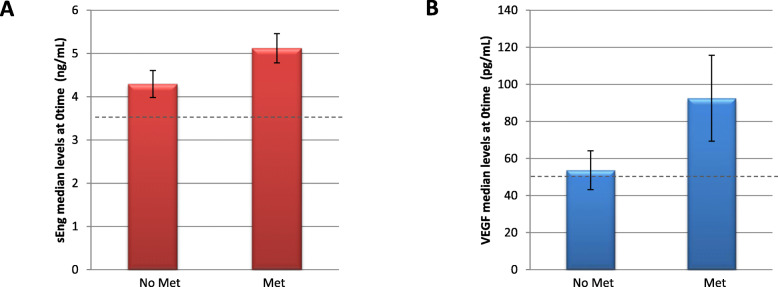
Soluble Endoglin and VEGF plasma levels comparing metastasis and non-metastasis groups of colon cancer. Plasma samples from different patients at time 0, at the same moment before resection of tumor sample were collected, and levels of sEng in nanograms per milliliter (**a**) and VEGF in picograms per milliliter (**b**) were measured by ELISA. The number of samples was *n* = 14 (non-metastasis VEGF group), *n* = 11 (metastasis VEGF group), *n* = 17 (non-metastasis sEng group), and *n* = 10 (metastasis sEng group). The dashed lines in both graphs indicate the considered normal values levels for each protein. sEng, soluble Endoglin; VEGF, Vascular Endothelial Growth Factor; Met, metastasis; No Met, non-metastasis

Focusing on sEng and VEGF levels during metastasis (Fig. 3) at time 0, patients with metastasis had slightly higher values of sEng (Fig. 3a), with a *p* value in the limit of significance (*p* = 0.0534). The VEGF levels before surgery were also higher, although not statistically significant (Fig. 3b).

### Soluble Endoglin (sEng) and VEGF plasma levels in patients with rectal cancer

**Fig. 4:**
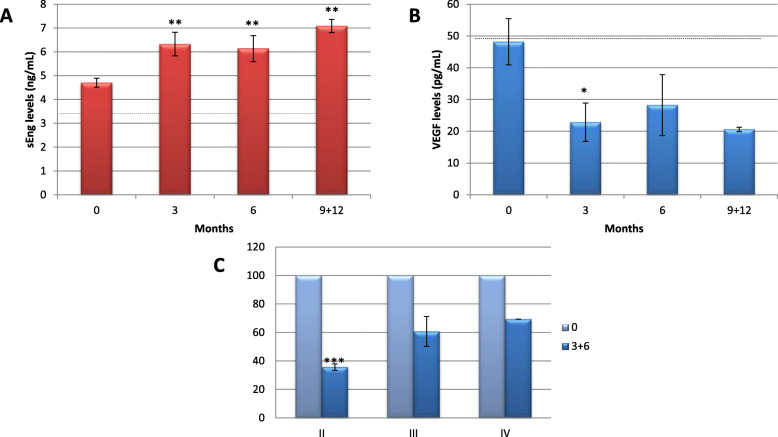
Soluble Endoglin and VEGF plasma levels change after resection of rectal tumor sample. Plasma samples from different patients at time 0 and at different time points (3, 6, and 9–12 months) after resection of tumor samples were collected, and levels of sEng in nanograms per milliliter (**a**) and VEGF in picograms per milliliter (**b**) were measured by ELISA and compared to time 0. Percentage of VEGF levels downregulated after resection of tumor and accompanied by a constant adjuvance treatment is represented in **c**, comparing different stages and time; the normalization is done between each time 0 in the specified stage. The number of samples was different between each time point: *n* = 28 (0), *n* = 18 (3), *n* = 7 (6), *n* = 7 (9 + 12) for sEng determination and *n* = 35 (0), *n* = 18 (3), *n* = 9 (6), *n* = 6 (9 + 12) for VEGF determination. Dashed lines in **a** and **b** graphs indicate the considered normal values levels for each protein. *p < 0.05; **p < 0.01 with respect to time 0 unless indicated between specified conditions. sEng, soluble Endoglin; VEGF, Vascular Endothelial Growth Factor

The 83.3% and 84% of patients with less or more aggressive RC, respectively, had higher than normal (3.5 ng/mL) mean sEng plasma levels at time 0 (Fig. 4a). Considering the evolution of these levels, sEng increases significantly over time. Due to the low number of samples, those obtained after 9 and 12 months were included in the same group.

Considering the TNM stage (Sup Figure 2A), sEng levels show a significant increase after resection in both less aggressive (*p* < 0.05) and more aggressive stages (*p* < 0.01).

Concerning VEGF, 41.7% and 46.2% of patients with less or more aggressive RC, respectively, had levels considered pathological at time 0, although the mean was in the borderline of the control value (below 30–50 pg/mL) (Fig. 4b). The VEGF levels tended to decrease during the follow-up period, according to the TNM stage. According to the TNM stage, VEGF decreases with time in less aggressive stages, while in more aggressive stages, VEGF levels remain low and similar (Sup Figure 2B).

In RC patients, insufficient data concerning the metastatic sample evolution of sEng and VEGF were obtained due to the limited sample number.

Interestingly, VEGF analysis of patients subjected to adjuvant treatment shows that VEGF is decreased after resection in all stages, but in more aggressive stages, the VEGF levels remain higher than in less aggressive stages (Fig. 4c).

### Endoglin protein in situ expression in colorectal tumors

The mRNA expression levels are not necessarily the same as the protein expression levels. Since an increase in sEng plasma levels in patients with CRC was found, we detected in situ Endoglin membrane expression by immunohistochemistry in tumoral versus non-tumoral tissue samples to correlate the sEng levels found in plasma. If there are blood vessels, we would expect Endoglin staining in the endothelium (inner part of the vessels); however, Endoglin is even expressed in the epithelial cells, in tumoral cells. The staining is specific, since controls with secondary antibody were made, and no staining was observed.

**Fig. 5:**
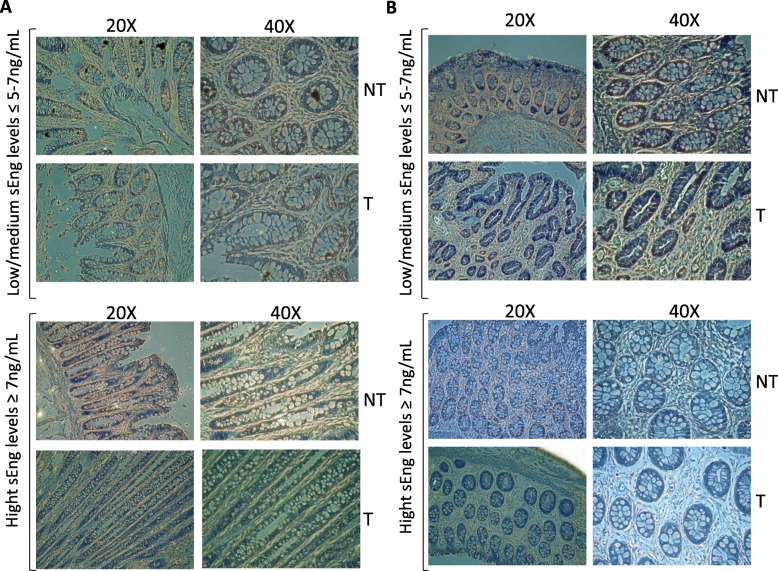
Endoglin immunostaining in colon and rectal cancer patient samples. One representative image of non-tumoral and tumoral zone from different patients differentiating between low/medium levels of sEng or high levels of sEng in plasma of these patients. Tumoral area (T) versus non-tumoral (NT) zone from colon (**a**) and rectal (**b**) cancer. Magnification × 20 and × 40

In Fig. 5, we can observe groups of 4 pictures, on the left side at × 20 magnification, on the right side at × 40 magnification. In the upper part, they correspond to non-tumoral tissue (NT), which was taken adjacent to the tumoral part during surgery.

Focusing on CC (Fig. 5a), we showed that in lower and medium plasma sEng levels, there were no staining differences between tumoral and non-tumoral counterparts in stroma and tubule cells. On the other hand, patients with higher sEng levels in plasma show more conspicuous Endoglin staining in the tumoral stroma, and there is no clear staining of tubules.

In the case of RC (Fig. 5b), in patients with lower sEng levels in plasma, Endoglin staining is similar in non-tumoral and tumoral areas, with the tubules and stroma stained almost at the same level of intensity. At higher levels of soluble Endoglin, staining in the tumoral stroma becomes fainter, and membrane Endoglin staining in the tubules practically disappears.

## Discussion

Colorectal cancer is an important problem regarding public health due to its high incidence and mortality. Its incidence is higher in men, and most of the patients are diagnosed above 50 years old [32]. In our study, 63.16% of patients were male, 36.84% were female, and 70.67% were older than 65 years old.

Currently, the prognosis and treatment depend on the clinic and histopathological stage, according to the TNM system [8]. Long-term survival depends equally on tumor stage, which is considered the most important factor related to mortality.

In the present study, we consider that colon and rectal cancer are different entities, given that they have a different embryological origin, anatomy, and functions [33]. Several biological and clinical hallmarks indicate that they are not the same entity; therefore, the treatments are different. Rectal cancer requires specific surgical treatment (total mesorectal excision, TME) preceded by neoadjuvant radiotherapy or chemoradiotherapy, depending on the location or stage, which reduces the risk of local recurrence, but does not improve survival compared to surgery alone. In colon cancer patients with positive lymph nodes, adjuvant systemic chemotherapy following curative surgery improves survival [34, 35].

Samples from both cancer types were analyzed for the expression of two putative biomarkers, Endoglin and VEGF. The expression at the RNA level of both genes was higher in tumoral tissues than in their non-tumoral counterparts. In CC, 80% of the tumors expressed higher levels of VEGF than those in normal tissues. This increased expression in tumor tissue suggests VEGF expression as a possible tumor marker.

Furthermore, VEGF was measured in plasma over time: preoperatively (immediately before surgery) and during followed up at 3, 6, and 9 months after resection of the tumor sample. Preoperative levels of VEGF in plasma were higher than the cut-off value (30–50 pg/mL). Our findings are similar to those previously published by the Nielsen group [36], in which VEGF levels in patients with CRC are higher than the values in healthy subjects. Interestingly, the average VEGF plasma levels in patients with CC were higher than the mean level in patients with RC (107.93 pg/mL versus 63.92 pg/mL, respectively). Although rectal tumors were diagnosed in more advanced stages and associated with a worse prognosis, these values may indicate different VEGF-dependent behavior between both types of tumors.

Comparing the different tumoral stages, more aggressive tumor stages corresponded to increased VEGF levels, well above the cut-off values of 30–50 pg/mL. Subsequently, we observed how after surgery, patients with less aggressive tumors showed a gradual decrease in VEGF levels over months, while patients with more aggressive tumors remained above normal values after 6 months. These data support some hypotheses described by other authors suggesting that persistent high levels of VEGF could be useful in predicting the radicality of CRC resection and could be applied as a prognostic marker [37–39].

Bevacizumab is an anti-angiogenic agent (targeting VEGF) that was administered to 80% of patients with metastatic colon cancer in our study, although the objective was not to analyze its impact on VEGF levels. Antiangiogenic agents in combination with cytotoxic chemotherapy are used to treat patients with metastatic CRC in first and second line; however, a large proportion of patients ultimately progress on bevacizumab therapy and other anti-VEGF therapies. The determination of VEGF plasma levels before and during of bevacizumab administration could be useful for its applicability, even to predict responses to treatment. Further studies with longer follow-up periods and a higher number of samples are required to clarify the importance of this biomarker [40].

Most of the studies concerning Endoglin and cancer have focused on its role as a proangiogenic factor and its utility as an MVD (micro-vessel density) marker. However, our study describes the relation between the specific membrane Endoglin staining and the sEng levels circulating in plasma. Moreover, Endoglin is even expressed in the epithelial cells; in tumoral cells, this is the important change to remark and the novelty of our study.

In CC, intermediate or low sEng levels in plasma are associated with no clear membrane Endoglin staining differences in stroma and tubules when comparing non-tumoral versus tumoral tissues. In contrast, when sEng levels are increased, the staining of membrane Endoglin is predominantly focused in tumor stroma (very vascularized tissue), and this staining disappears in the tubules. This effect is probably due to the active MMP14 shedding of membrane Endoglin and its release into the blood, when becoming more aggressive.

In RC, when sEng levels were low, there was predominant and specific staining of Endoglin in the tubule membrane (or Lieberkühn’s tubular vaults) of non-tumoral areas and to a lesser extent in the tumor stroma. As sEng levels increase, membrane Endoglin staining is predominant in the tumor stroma area and finally disappears when sEng levels are high, which is normally concurrent with high-grade tumoral stages. This correlation could again be explained because membrane Endoglin is predominantly expressed in endothelial cells, which are localized in the stroma, and its expression may help to increase the tumoral angiogenesis process. Endoglin expression in tumor cells decreases over progression because processes such as migration, invasion, and epithelial mesenchymal transition are taking place, and the metalloprotease MMP14, which is responsible for the shedding of membrane Endoglin, is active and increases sEng release into the medium [23, 24]. Therefore, Endoglin expression changes in the tumor and in the vascular endothelium, modulating malignancy [40].

The fact that high sEng levels in patients are associated with poor cancer prognosis is interesting. A possible explanation could derive from its origin, since sEng production might be a late event in carcinogenesis progression, and its antiangiogenic function may be irrelevant to tumor growth [41] in the most advanced stages.

Moreover, sEng preoperative levels were found to be higher than normal values in 83.8% and 79% of patients with RC or CC, respectively. Although no statistically significant differences were found, there was a clear tendency of higher sEng levels in more aggressive stages than in less aggressive stages [42–45]. Strikingly, preoperative sEng levels between patients with and without metastasis showed a statistically significant difference in RC, with 87.91 ng/mL versus 4.70, respectively, consistent with the same references mentioned above.

This study is limited by the number of samples corresponding to each tumor stage and the difficulty in obtaining all the necessary samples. Another important issue that limits the consistency of the study is the fact of not having samples from a control group without evidence of disease. For this reason, a second phase of the study with a higher number of patients would expand the results favorably and allow more consistent conclusions.

## Conclusions

Considering our results, we conclude that VEGF gene expression is normally higher in tumor tissue and therefore can be considered a good angiogenesis tumor marker in CRC. VEGF can also be an evolutionary indicator of CRC patients after surgical intervention, taking into account that in some cases, therapy can interfere with this effect. The two forms of Endoglin, membrane and soluble, can also be used to monitor the tumor samples. The membrane form is related to less or more aggressive stages, whereas soluble Endoglin levels can be used to monitor the first signs of metastasis.

## Supplementary information

**Additional file 1: Figure S1**. Soluble endoglin and VEGF plasma levels considering colon cancer TNM stage and time. Plasma samples from different patients at time 0 and at different time points (3, 6 and 9-12 months) after resection of tumor samples were collected. Those samples were divided considering TNM stages in less aggressive tumors (malignant polyp and stages 0, I and II) and more aggressive tumors (stage III and IV). Levels of sEng in ng/mL (A) and VEGF in pg/mL (B) were measured by ELISA and compared to time 0. The dashed lines in both graphs indicate the considered normal values levels for each protein. The number of samples were different between each time point: n=23 (0), n=9 (3+6), n=5 (9+12) for sEng and stages I and II; n=22 (0), n=19 (3+6), n=11 (9+12) for sEng and stages III and IV; n=23 (0), n=9 (3+6), n=5 (9+12) for VEGF and stages I and II; n=22 (0), n=21 (3+6), n=11 (9+12) for VEGF and stages III and IV. *p<0.05 with respect to time 0 unless indicated between specified conditions. sEng: soluble endoglin; VEGF: Vascular Endothelial Growth Factor. **Figure S2**. Soluble endoglin and VEGF plasma levels considering rectal cancer TNM stage and time. Plasma samples from different patients at time 0 and at different time points (3, 6, 9 and 12 months) after resection of tumour samples were collected. Those samples were divided considering TNM stages in less aggressive tumours (stage I+II) and more aggressive tumours (Stage III+IV). Levels of sEng in ng/mL (A) and VEGF in pg/mL (B) were measured by ELISA and compared to time 0. The dashed lines in both graphs indicate the considered normal values levels for each protein. The number of samples were different between each time point: n=8 (0), n=6 (3+6), for sEng and stages I and II; n=18 (0), n=16 (3+6) for sEng and stages III and IV; n=7 (0), n=5 (3+6) for VEGF and stages I and II; n=18 (0), n=18 (3+6) for VEGF and stages III and IV. *p<0.05; **p<0.01 with respect to time 0. sEng: soluble endoglin; VEGF: Vascular Endothelial Growth Factor.

## Data Availability

All data utilized in this study are not public but are available from the corresponding authors upon reasonable request.

## Abbreviations

AJCC: American Joint Committee on Cancer
CC: Colon cancer
CRC: Colorectal cancer
CT: Computed tomography
Eng: Endoglin
MR: Magnetic resonance
MVD: Micro-vessel density
RC: Rectal cancer
sEng: Soluble form of Endoglin
TGF-β: Transforming Growth Factor-β
TME: Total mesorectal excision
VEGF: Vascular Endothelial Growth Factor
UICC: Union for International Cancer Control
