# Supplementary figures and images for: Endoglin (CD105) and VEGF as potential angiogenic and dissemination markers for colorectal cancer
Source: World J Surg Oncol. 2020 May 20;18:99. doi: 10.1186/s12957-020-01871-2 (PMC7240983; doi:10.1186/s12957-020-01871-2)

## Slide 1
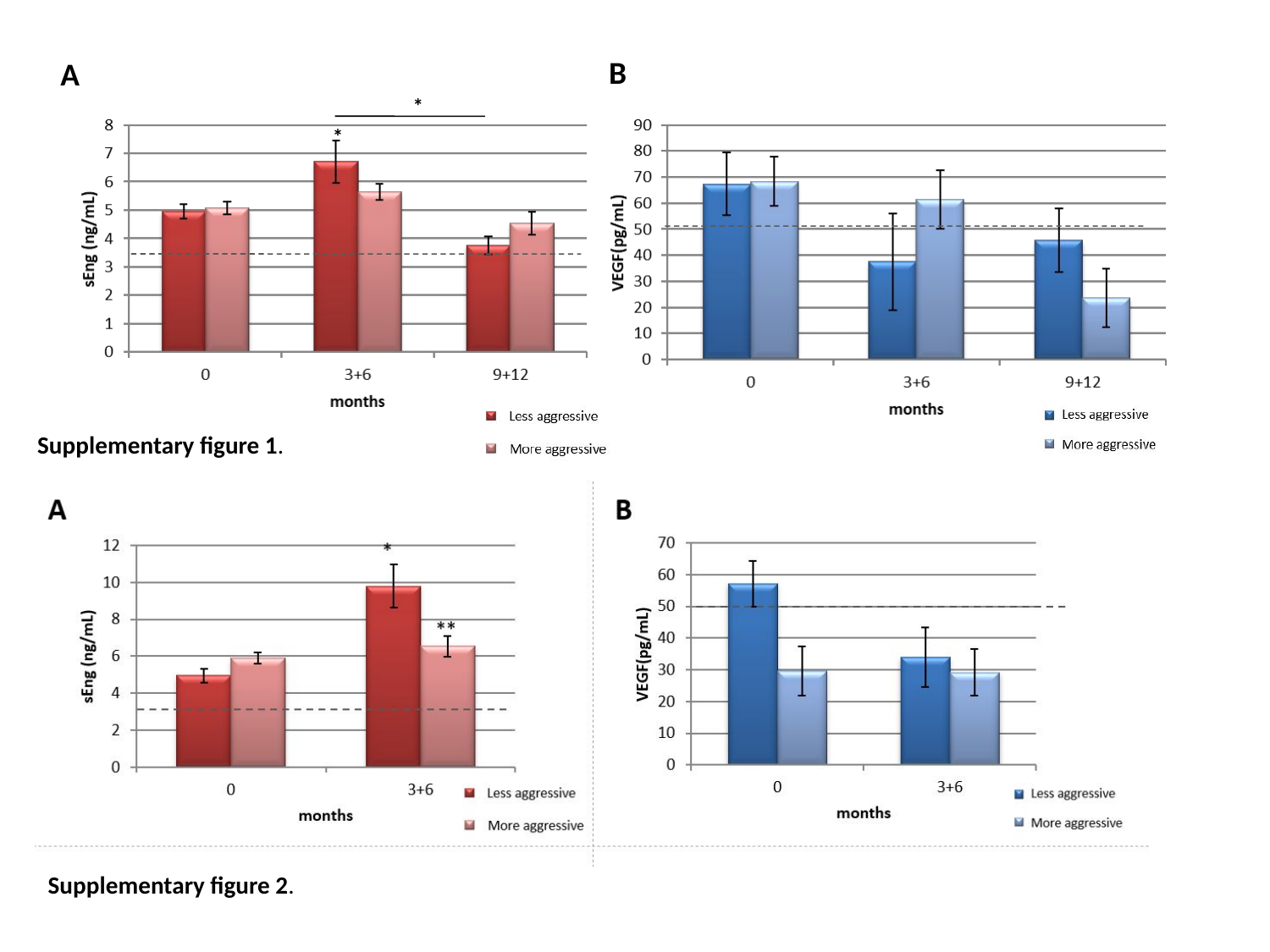

B
A
*
*
Supplementary figure 1.
Supplementary figure 2.

Supplement: Supplementary file 1 — Additional file 1: Figure S1. Soluble endoglin and VEGF plasma levels considering colon cancer TNM stage and time. Plasma samples from different patients at time 0 and at different time points (3, 6 and 9-12 months) after resection of tumor samples were collected. Those samples were divided considering TNM stages in less aggressive tumors (malignant polyp and stages 0, I and II) and more aggressive tumors (stage III and IV). Levels of sEng in ng/mL (A) and VEGF in pg/mL (B) were measured by ELISA and compared to time 0. The dashed lines in both graphs indicate the considered normal values levels for each protein. The number of samples were different between each time point: n=23 (0), n=9 (3+6), n=5 (9+12) for sEng and stages I and II; n=22 (0), n=19 (3+6), n=11 (9+12) for sEng and stages III and IV; n=23 (0), n=9 (3+6), n=5 (9+12) for VEGF and stages I and II; n=22 (0), n=21 (3+6), n=11 (9+12) for VEGF and stages III and IV. *p<0.05 with respect to time 0 unless indicated between specified conditions. sEng: soluble endoglin; VEGF: Vascular Endothelial Growth Factor. Figure S2. Soluble endoglin and VEGF plasma levels considering rectal cancer TNM stage and time. Plasma samples from different patients at time 0 and at different time points (3, 6, 9 and 12 months) after resection of tumour samples were collected. Those samples were divided considering TNM stages in less aggressive tumours (stage I+II) and more aggressive tumours (Stage III+IV). Levels of sEng in ng/mL (A) and VEGF in pg/mL (B) were measured by ELISA and compared to time 0. The dashed lines in both graphs indicate the considered normal values levels for each protein. The number of samples were different between each time point: n=8 (0), n=6 (3+6), for sEng and stages I and II; n=18 (0), n=16 (3+6) for sEng and stages III and IV; n=7 (0), n=5 (3+6) for VEGF and stages I and II; n=18 (0), n=18 (3+6) for VEGF and stages III and IV. *p<0.05; **p<0.01 with respect to time 0. sEng: soluble endoglin; VEGF: Vas [file 12957_2020_1871_MOESM1_ESM.ppt]
